# Cerebral infarction detected after laparoscopic partial hepatectomy: case report

**DOI:** 10.1186/s40981-019-0301-7

**Published:** 2019-12-12

**Authors:** Atsushi Kobayashi, Kazuhiro Shirozu, Yuji Karashima, Katsuyuki Matsushita, Ken Yamaura

**Affiliations:** 1 Department of Anesthesiology and Critical Care Medicine, Kyushu University Hospital, 3-1-1 Maidashi, Higashi-ku, Fukuoka, 812-8582 Japan; 2 Department of Anesthesiology and Critical Care Medicine, Graduate School of Medical Sciences, Kyushu University, Fukuoka, Japan; 3 Operating Rooms, Kyushu University Hospital, Fukuoka, Japan

**Keywords:** Brain infarction, Laparoscopic hepatectomy, Patent foramen ovale

## Abstract

**Background:**

Bleeding and carbon dioxide (CO_2_) gas embolism have been reported as serious complications associated with laparoscopic surgery. We present a case of cerebral infarction presumably caused by CO_2_ gas embolism during laparoscopic hepatectomy.

**Case presentation:**

During liver resection, the end-tidal CO_2_ suddenly dropped from 40 to 21 mmHg. Simultaneously, ST elevation in lead II and ST depression in lead V5 of the electrocardiogram were observed. After improvement of these electrocardiographic changes, surgery was continued. Postoperatively, incomplete paralysis was present in the right arm and leg. Magnetic resonance imaging revealed cerebral infarction in the broad area of the left cerebral cortex. These complications might have been caused by paradoxical embolism.

**Conclusion:**

We should always keep in mind the risk of cerebral infarction with neurological deficits in the case of laparoscopic surgery. Careful monitoring and appropriate treatment for gas embolism are necessary during laparoscopic surgery.

## Background

Laparoscopic liver resection has recently been recognized as a safe surgical procedure for liver disease and has gradually become more widespread. Carbon dioxide (CO_2_) is generally used for pneumoperitoneum. Laparoscopic surgery has led to a reduction in the postoperative length of hospital stay. However, bleeding and CO_2_ gas embolism have been reported as serious complications associated with this procedure [1, 2].

The incidence of gas embolism is rare, approximately 0.15% for laparoscopic surgery and 0.2 to 1.5% for laparoscopic hepatectomy [3]. Its most common cause is the entrapment of CO_2_ within an injured vein or solid organ [4, 5]. A serious complication is paradoxical embolism which is due to the migration of venous emboli into the arterial circulation through an arteriovenous shunt [6, 7] with resultant cerebral and myocardial infarction. We herein present a case of cerebral infarction presumably caused by paradoxical embolism during laparoscopic hepatectomy.

## Case presentation

A 61-year-old man (height, 157 cm; weight, 56 kg) was diagnosed with recurrent hepatocellular carcinoma with cirrhosis in the right hepatic lobe and was scheduled for laparoscopic hepatectomy. He had no clinical cardiopulmonary or brain disease.

The operation was performed under combined general and epidural anesthesia. Anesthesia was induced with propofol 70 mg and fentanyl 100 μg. After administration of rocuronium 30 mg, tracheal intubation was performed. Anesthesia was maintained with desflurane, a 40 to 50% oxygen–air mixture, and continuous infusion of remifentanil 0.15 μg × kg^−1^ × min^−1^. The arterial blood pressure, pulse oximetry, capnography, and central venous pressure were monitored continuously. Volume-controlled ventilation was adopted to obtain an end-tidal CO_2_ (EtCO_2_) level of 30 to 40 mmHg.

The patient was placed in the head-down position during surgery, and intra-abdominal pressure was maintained to 10 cmH2O. During the pneumoperitoneum, the tidal volume was adjusted to 500 ml, and the respiratory rate was adjusted to maintain the EtCO_2_ at 30 to 40 mmHg. The fluid management strategy was adjusted to maintain CVP about 5 mmHg.

**Fig. 1:**
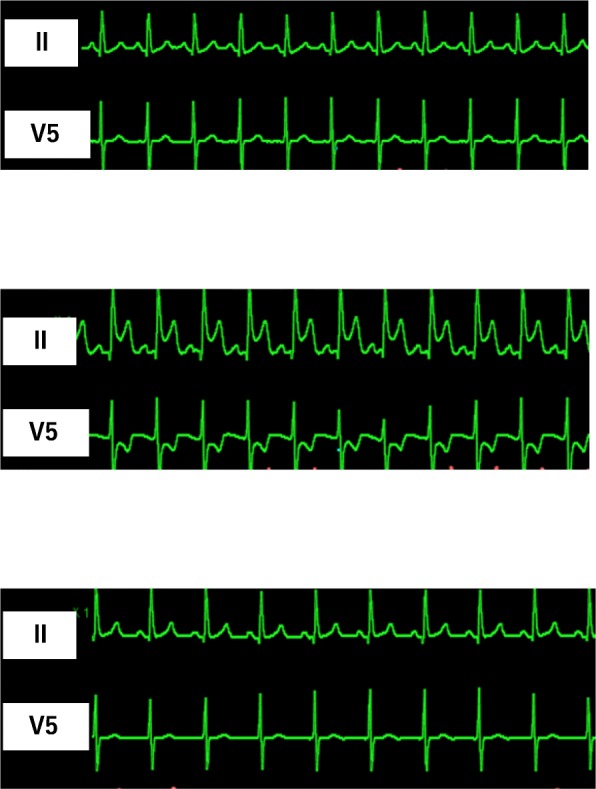
Electrocardiogram in leads II and V5 before (upper), during (middle), and after (lower) ST change

Laparoscopic hepatic resection was started after clamping the branches of the vascular pedicle, commonly known as the Pringle maneuver. During the hepatic resection, the EtCO2 dropped from 40 to 21 mmHg, and ST elevation was observed in lead II and ST depression was observed in lead V5 of the electrocardiogram (Fig. 1, middle). At this time, the electrocardiogram showed sinus rhythm and the blood pressure was almost stable; however, the central venous pressure slightly increased from 7 to 13 mmHg. After the operation was stopped, arterial blood gas analysis showed a divergence between the partial pressure of CO_2_ (67.4 mmHg) and EtCO_2_ (21 mmHg). Although gas embolism was strongly suspected, coronary artery spasm could not be ruled out because the ST change in leads II and V5 was suspected to be associated with the right coronary artery. The electrocardiographic changes improved shortly thereafter (Fig. 1, lower), and the surgery was resumed after administration of a coronary vasodilator (nicorandil, 3 mg/h) and β-blocker (landiolol, 5 mg). The surgery was completed without further problems.

**Fig. 2:**
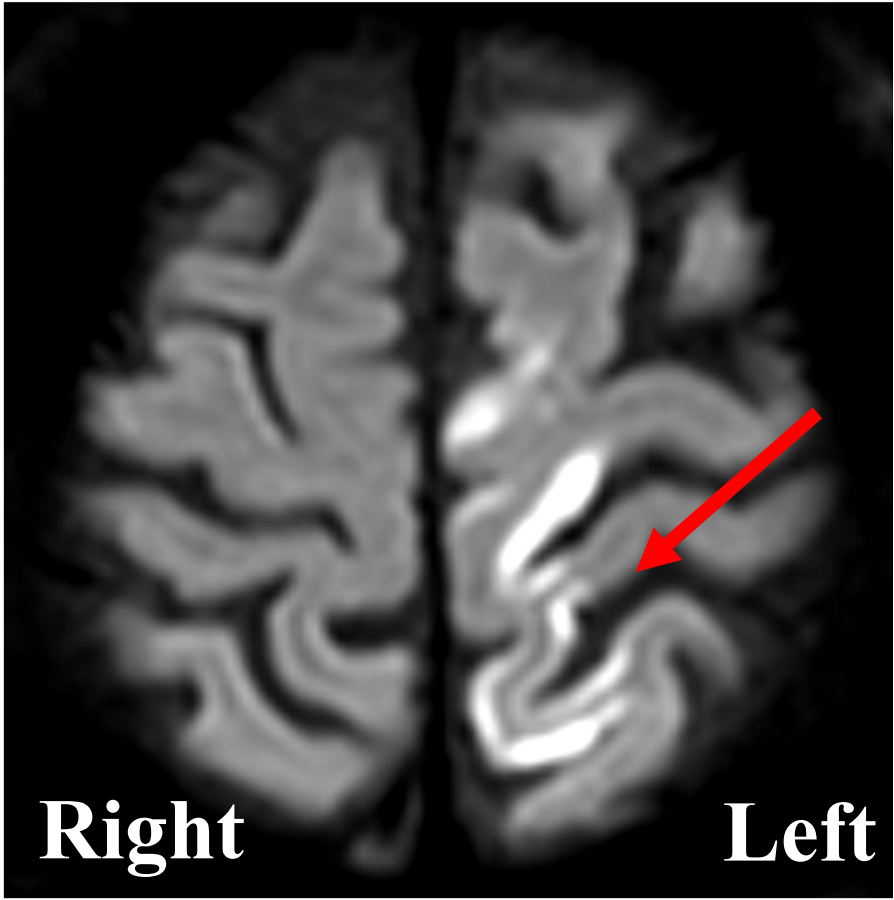
Postoperative T2-weighted magnetic resonance imaging of the brain. The red arrow indicates a high-density presence at the left frontal lobe

After the surgery, the patient’s hemodynamic state was stable and tracheal extubation was performed smoothly 20 min postoperatively. Although the patient could speak and had alert consciousness, he exhibited muscle weaknesses in the right arm and leg. Obvious cerebral bleeding was not detected by emergency computed tomography immediately after surgery; however, magnetic resonance imaging performed the next day revealed cerebral infarction in the broad area of the left cerebral cortex (Fig. 2). Transthoracic echocardiography was performed to determine the cause of the cerebral infarction, but no intracardiac defects were found. The patient was able to walk independently with a cane and was discharged 21 days after the surgery.

## Discussion

Although gas embolism is a rare complication, cerebral infarction by gas embolism is particularly rare. According to a systematic literature review, 3 cases (0.2%) of gas embolism were reported among 1262 patients who underwent laparoscopic major hepatectomy [8]. The reported mortality rate of CO_2_ embolism is 28% [9]. Once CO_2_ enters the systemic venous system, it is transported to the lungs and CO_2_ embolism occurs, resulting in cardiac arrhythmias, pulmonary hypertension, and hypotension [5]. In comparison with general laparoscopic surgery, the hepatic vein could be sufficiently exposed on the surface to be transected during anatomical resection in major hepatectomy [8, 10]. Thus, CO_2_ embolism might more frequently occur in laparoscopic hepatectomy than in the other laparoscopic surgeries.

Paradoxical embolism provokes systemic embolism caused by the presence of an intracardiac defect, intrapulmonary arteriovenous shunt, or pulmonary fistula [2, 6, 7, 11]. Intrapulmonary shunts may occur in patients with direct arteriovenous communications and dilated pre-capillaries in the lungs secondary to liver cirrhosis or pulmonary arteriovenous malformation [2]. Because almost all cerebral gas embolisms are diagnosed after observation of delayed recovery from general anesthesia or impaired consciousness, the diagnosis of paradoxical embolism during surgery is difficult [1, 7].

A patent foramen ovale (PFO) is found in approximately 25% of healthy individuals [12]. However, a PFO is usually closed under normal physiological conditions. Thus, detection of a small PFO before and after surgery is difficult because opening of the PFO and right-to-left shunting mainly occur when the right atrial pressure exceeds the left atrial pressure [12]. Transesophageal echocardiography (TEE) is considered the gold standard for diagnosis because it allows for direct anatomic visualization of shunting [12]. Actually, TEE can reveal the existence of the PFO in approximately 24% of patients in contrast to transthoracic echocardiography (15% of patients). However, an opened PFO is not necessarily observed by TEE with manual positive-pressure ventilation during surgery. In the present case, we should have examined the patient for an air embolism using TEE when the ST change developed during surgery.

The reason for the brain infarction in the present case is unclear, but we considered three possibilities. The first possibility is that the right atrial pressure exceeded the left atrial pressure secondary to the CO_2_ embolism caused right-to-left shunting through an open PFO. This possibility could also explain the ST change in the electrocardiogram. The second possibility is the development of low blood pressure. The minimum intraoperative blood pressure was 51/24 mmHg and the duration of time for which the systolic pressure was < 60 mmHg was about 3 min. However, it seems that this hypotension period was not clinically important. The third possibility is the development of an intrapulmonary shunt caused by liver cirrhosis. Such shunts develop in 15 to 45% of patients with liver cirrhosis [2]. Kawahara et al. [2] reported the occurrence of cerebral infarction during laparoscopic surgery. They concluded that the cause was an intrapulmonary shunt because an intracardiac shunt was not observed by TEE.

The mechanism of ST change in this case could have been a gas embolism or coronary spasm. However, the present report indicates that an ST change on the electrocardiogram could be helpful when paradoxical embolism is suspected. Additionally, if we had acted quickly to reduce the risk of paradoxical air embolism (i.e., moving the patient to the Trendelenburg position, initiating TEE, changing the procedure to open surgery, or suctioning air from the right atrium via central venous catheter if present) when the ST change developed in this case, the cerebral infarction might have been prevented. Thus, we suggest preoperative TTE examination for detection of PFO and intraoperative monitoring of the bispectral index or regional oxygen saturation which could possibly detect gas embolism [2]. TEE is reportedly the most sensitive technique for detecting intracardiac bubbles [2, 5]. However, we believe that TEE should be used for high-risk patients who are expected to have a large amount of bleeding. Furthermore, an adequate central venous pressure should be maintained, and any increase in the intra-abdominal pressure during laparoscopic hepatectomy should be addressed.

## Conclusion

We should always keep in mind the risk of cerebral infarction with neurological deficits in the case of laparoscopic surgery in which surgery time is long or bleeding amount is large. Careful monitoring such as TEE or the bispectral index, and appropriate treatment for gas embolism, including cerebral infarction, is necessary during laparoscopic surgery.

## Data Availability

None

## Abbreviations

CO_2_: Carbon dioxide
EtCO_2_: End-tidal carbon dioxide
PFO: Patent foramen ovale
TEE: Transesophageal echocardiography

## References

[1] Shin HY, Kim DW, Kim JD, Yu SB, Kim DS, Kim KH, Ryu SJ (2014). Paradoxical carbon dioxide embolism during laparoscopic cholecystectomy as a cause of cardiac arrest and neurologic sequelae: a case report. Korean J Anesthesiol.

[2] Kawahara T, Hagiwara M, Takahashi H, Tanaka M, Imai K, Sawada J, Kunisawa T, Furukawa H (2017). Cerebral infarction by paradoxical gas embolism during laparoscopic liver resection with injury of the hepatic vessels in a patient without a right-to-left systemic shunt. Am J Case Rep.

[3] Kim CS, Liu J, Kwon JY, Shin SK, Kim KJ (2008). Venous air embolism during surgery, especially cesarean delivery. J Korean Med Sci.

[4] Scoletta P, Morsiani E, Ferrocci G, Maniscalco P, Pellegrini D, Colognesi A (2003). Azzena G: [Carbon dioxide embolization: is it a complication of laparoscopic cholecystectomy? ]. Minerva Chir.

[5] Park EY, Kwon JY, Kim KJ (2012). Carbon dioxide embolism during laparoscopic surgery. Yonsei Med J.

[6] Geng J, Tian HY, Zhang YM, He S, Ma Q, Zhang JB, Liu Y, Tian H, Zhang D, Meng Y (2017). Paradoxical embolism: a report of 2 cases. Medicine (Baltimore).

[7] Windecker S, Stortecky S, Meier B (2014). Paradoxical embolism. J Am Coll Cardiol.

[8] Otsuka Y, Katagiri T, Ishii J, Maeda T, Kubota Y, Tamura A, Tsuchiya M, Kaneko H (2013). Gas embolism in laparoscopic hepatectomy: what is the optimal pneumoperitoneal pressure for laparoscopic major hepatectomy?. J Hepatobiliary Pancreat Sci.

[9] Cottin V, Delafosse B, Viale JP (1996). Gas embolism during laparoscopy: a report of seven cases in patients with previous abdominal surgical history. Surg Endosc.

[10] Foo E, Williams D, Singh H, Bridgman PG, McCall J, Connor S (2012). Successful management of a large air embolus during an extended right hepatectomy with an emergency cardiopulmonary bypass. HPB (Oxford).

[11] Sendt W, Schummer W, Altendorf-Hofmann A, Weber T (2009). Paradoxical carbon dioxide embolism during laparoscopic unroofing of a recurrent nonparasitic liver cyst. Can J Surg.

[12] Miranda B, Fonseca AC, Ferro JM (2018). Patent foramen ovale and stroke. J Neurol.

